# Theoretical insights into the methane catalytic decomposition on graphene nanoribbons edges

**DOI:** 10.3389/fchem.2023.1172687

**Published:** 2023-06-01

**Authors:** Neubi F. Xavier, Anthony J. R. Payne, Glauco F. Bauerfeldt, Marco Sacchi

**Affiliations:** ^1^ School of Chemistry and Chemical Engineering, University of Surrey, Guildford, United Kingdom; ^2^ Instituto de Química, Universidade Federal Rural Do Rio de Janeiro, Seropédica, Brazil

**Keywords:** methane, DFT, carbon catalysis, graphene edges and nanoribbons, hydrogen

## Abstract

Catalytic methane decomposition (CMD) is receiving much attention as a promising application for hydrogen production. Due to the high energy required for breaking the C-H bonds of methane, the choice of catalyst is crucial to the viability of this process. However, atomistic insights for the CMD mechanism on carbon-based materials are still limited. Here, we investigate the viability of CMD under reaction conditions on the zigzag (12-ZGNR) and armchair (AGRN) edges of graphene nanoribbons employing dispersion-corrected density functional theory (DFT). First, we investigated the desorption of H and H_2_ at 1200 K on the passivated 12-ZGNR and 12-AGNR edges. The diffusion of hydrogen atom on the passivated edges is the rate determinant step for the most favourable H_2_ desorption pathway, with a activation free energy of 4.17 eV and 3.45 eV on 12-ZGNR and 12-AGNR, respectively. The most favourable H_2_ desorption occurs on the 12-AGNR edges with a free energy barrier of 1.56 eV, reflecting the availability of bare carbon active sites on the catalytic application. The direct dissociative chemisorption of CH_4_ is the preferred pathway on the non-passivated 12-ZGNR edges, with an activation free energy of 0.56 eV. We also present the reaction steps for the complete catalytic dehydrogenation of methane on 12-ZGNR and 12-AGNR edges, proposing a mechanism in which the solid carbon formed on the edges act as new active sites. The active sites on the 12-AGNR edges show more propensity to be regenerated due lower free energy barrier of 2.71 eV for the H_2_ desorption from the newly grown active site. Comparison is made between the results obtained here and experimental and computational data available in the literature. We provide fundamental insights for the engineering of carbon-based catalysts for the CMD, showing that the bare carbon edges of graphene nanoribbons have performance comparable to commonly used metallic and bi-metallic catalysts for methane decomposition.

## 1 Introduction

In the last 3 decades, catalytic methane decomposition (CMD) has received great attention as a promising highly-efficient hydrogen production process (Pinaeva et al., 2017; Alves et al., 2021; Fan et al., 2021; Hamdan et al., 2022). Much of the interest in this process is motivated by the promising potential of the ‘turquoise’ (Hermesmann and Müller, 2022) hydrogen obtained by CMD to replace fossil fuels, without obtaining CO_*x*_ as by-products (Pomerantseva et al., 2019; Russell et al., 2021; Hamdan et al., 2022; Jiang et al., 2022; Yang and Gao, 2022). The research effort regarding CMD has dramatically increased since the beginning of the 1990s (Alves et al., 2021), however only techniques like autothermal reforming (ATR), steam methane reforming (SRM), dry reforming of methane (DRM) and partial oxidation (POX) into synthesis gas (also called ‘syngas’ - consisting in a mixture of CO and H_2_) have been shown maturity for industrial applications (Pinaeva et al., 2017; Tong et al., 2022). In fact, SMR accounts for more than 90% of the global H_2_ supply, leading to an emission of 830 Mt of CO_2_ per year (Tong et al., 2022). Therefore, low-temperature cracking of methane performed through CMD is a promising solution to tackle CO_*x*_ production, since its main products are pure hydrogen gas and solid carbon (CH_4_ → 2H_2_ + C; ΔH = 75 kJ mol^−1^) (Alves et al., 2021; Yang and Gao, 2022).

The main challenge of CMD is that methane is a very inactive precursor with a strong C-H bond energy of 440 kJ mol^−1^. For this reason, the most widely employed methane cracking processes require very high temperatures (above 1473 K) (Abánades et al., 2016; Qian et al., 2020) and the usage of a metal-based catalyst is necessary to achieve lower temperatures (773 K-1073 K) for efficient conversion (Ashik et al., 2015). In this aspect, considerable efforts have been made in designing more efficient and sustainable heterogeneous catalysts for methane cracking. Currently, metal-based catalysts are the most commonly employed for this reaction and, among those, nickel and iron-based catalysts stand out for their economic viability, practicality and for possessing high selectivity to produce hydrogen from methane decomposition (Reshetenko et al., 2004; Fan et al., 2021; Hamdan et al., 2022; Rattanaamonkulchai et al., 2022). On the other hand, as other metal catalysts, their performance suffers from rapid deactivation by carbon poisoning during the CMD (Hadian et al., 2021; Jiang et al., 2022; Rattanaamonkulchai et al., 2022; Yan et al., 2022).

Graphitic carbon materials such as graphite (3D) and carbon nanotubes (1D) have been investigated as alternative materials for methane cracking since they have higher resistance to carbon poisoning (Hamdan et al., 2022; Yang and Gao, 2022). For the latter, recent works (Wang et al., 2021; Rattanaamonkulchai et al., 2022) have reported carbon growth on the catalyst surface alongside H_2_ production by methane decomposition (Chai et al., 2006; Ni et al., 2006; Pudukudy et al., 2018; Esteves et al., 2020). Among the novel carbon-based materials that have been investigated as CMD catalysts, graphene has generated considerable interest (Suelves et al., 2008; Guil-Lopez et al., 2011; Szymańska et al., 2015). Graphene is the 2D monolayer of graphite and is the fundamental building block for other carbon allotropes (Geim and Novoselov, 2007). The catalytic performances of graphene can be improved by tuning its surface properties, e.g., by heteroatomic substitution (Rao et al., 2014; Lawrence et al., 2021), adatoms (Castro Neto et al., 2009; Pizzochero and Kaxiras, 2022), defects (Han et al., 2019; Brooks et al., 2022) and inclusion of functional groups (Kuila et al., 2012; Wood et al., 2012; He et al., 2022). Graphene nanoribbon (GNR) are < 10 nm wide strips of graphene and can be obtained by cutting the graphene layer in one specific dimension (Li et al., 2008; Kosynkin et al., 2009; Dutta and Pati, 2010). The properties of GNRs are mainly defined by their edges (Jia et al., 2011; Fujii and Enoki, 2013), making them tunable and promising materials for catalysis (Zhang et al., 2018; Peng et al., 2021), for sensors (Wood et al., 2012; Suman et al., 2020; He et al., 2022) and all-carbon spintronics (Pizzochero and Kaxiras, 2022).

The CMD mechanism is expected to be initiated by the adsorption and dissociation of methane molecules on the catalyst active sites, followed by a series of surface deprotonation reactions. However, there is a considerable lack of agreement regarding the viability of the main decomposition mechanism and relevant intermediates structures on carbonaceous catalysts (Zhang et al., 2017; Fan et al., 2021). Although atomistic insights for methane cracking on metallic and bi-metallic catalysts have been widely reported in the literature (Li et al., 2014; Calderón et al., 2016; Arevalo et al., 2017; Salam and Abdullah, 2017; Palmer et al., 2019), the reaction mechanism of CMD on different surface structures (e.g., free valence sites, edges and vacancies) of carbon-based catalysts, elucidating the hydrogen formation channels, are limited to the reverse steps of the methanation reactions (Calderón et al., 2016; Zhang et al., 2017; Fan et al., 2021). To the best of our knowledge, this is the first time that the methane decomposition steps are investigated on graphene edges through first-principles methodologies under the reaction conditions of the CMD process. In this work, we presented a computational investigation of graphene nanoribbons edges as a catalyst for the CMD. We started our investigation by analysing the dehydrogenation mechanisms over the two distinct edge morphologies: zigzag nanoribbons (12-ZGNRs) and armchair nanoribbons (12-AGNRs). We report the Gibbs free energy profile for the formation of bare carbon active sites at 1200 K. We analyse of the CH_4_ reactivity on the edges starting by the physisorption of methane on the zigzag and armchair edges. After, we focused on the mechanism of methane decomposition on 12-ZGNRs and 12-AGNRs, i.e., the deprotonation steps and H_2_ formation. Our results were compared with literature reports, aiming to provide a full assessment of GNRs as catalysts for methane dissociation and hydrogen evolution and to provide insights for future works on the catalyst engineering of graphene-based materials. In our previous work (Xavier et al., 2023), we found that decoration of non-metallic heteroatoms on the nanocarbons edges can dramatically increase the regeneration of carbonaceous catalysts, however, the performance for methane decomposition reported here on the pure carbon edges was found to be superior. We expect that the insights provided here aid the engineering of carbon-based catalysts for the catalytic methane decomposition.

## 2 Computational details

In this work, calculations were carried out adopting periodic boundary conditions, within the density functional theory (DFT) framework, as implemented in the CASTEP package (Segall et al., 2002; Clark et al., 2005). The generalized gradient approximation (GGA) exchange-correlation functional devised by Perdew, Burke and Ernzerhof (Perdew et al., 1996) was adopted. Core electrons of atoms were treated by ultrasoft pseudopotentials of Vanderbilt (Vanderbilt, 1990). Non-covalent interactions were accounted for through the adoption of the TS dispersion correction method (Tkatchenko and Scheffler, 2009). The more robust Many-Body Dispersion correction scheme (Tkatchenko et al., 2012) was adopted in selected cases, for comparison purpose, and discussed throughout the manuscript. Convergence tests of the kinetic energy cut-off and k-point sampling were made and a value of 550 eV and a 2 × 1 × 1 Monkhorst-Pack (Monkhorst and Pack, 1976) grid were adopted, respectively. A geometry optimization scheme based on the Broyden-Fletcher-Goldfarb-Shanno (BFGS) algorithm (Pfrommer et al., 1997) was adopted, as implemented in CASTEP. Transition states were obtained by adopting the Linear-Quadratic-Synchronous Transit (LST/QST) algorithm (Govind et al., 2003). In this double-ended methodology, the starting points for the calculations are the reactants and products of each reaction step. Therefore, the workflow for locating the transition states is to first perform the optimization of the local minima points under a force tolerance of 0.025 eV Å^−1^ and SCF energy tolerance of 1 × 10–^6^ eV. In the second step of the workflow, we adopt the previously optimized stationary points as starting structures for the LST/QST calculation. Transition states were confirmed by the presence of a single imaginary frequency respective to the reaction coordinate in the vibrational analysis. The surface, adsorbate and isolate molecules were allowed to move during geometry optimisation and transition state searches. Adsorption energies (*E*_*ads*_) were estimated as shown in Eq. 1.

$$E_{\mathrm{ads}}=E_{g+GNR}-E_{\mathrm{GNR}}-E_{g}$$ (1)

where *E*_*.g.,*_ is the total energy of the isolated gas species. The *E*_*GNR*_ and *E*_*.g.,*+*GNR*_ terms are related to graphene nanoribbons and the adsorbed system, respectively. The gas-phase species investigated here, i.e., CH_4_, H_2_ and the isolated hydrogen atom, H, were assumed as reference for the calculation of adsorption energies. Vibrational properties were obtained by phonons calculations at the Γ-point adopting the partial Hessian vibrational analysis (Li and Jensen, 2002). In this approach, we only considered the normal modes of the adsorbate and the two atomic rows closest to adsorbate and low-frequency vibrational modes were treated as 200 cm^−1^, similar to the approach adopted in our previous work (Xavier et al., 2023). The partition functions of the adsorbates were estimated with the lattice gas approach. (Campbell et al., 2016; Knopf and Ammann, 2021) The Gibbs free energy was calculated for the reaction conditions of 1200 K and 1 bar with the fundamental equation *G* = *H* − *TS*, in which *H* is the enthalpy, comprising the DFT energy, the zero-point energy correction and thermal contributions, and *S* is the entropy. For the gas-phase molecule, the translational, rotational and vibrational partition functions were considered, accordingly to conventional statistical thermodynamic expressions. The isolated molecules were optimised inside a box of 20 Å and we assumed the experimental vibrational data retrieved from the NIST database (Johnson et al., 2020). The Gibbs free energy of activation was estimated as Δ*G*_*a*_ = *G*_*TS*_ − *G*_*IS*_, in which *G*_*TS*_ is the free energy of the transition state and *G*_*IS*_ is the free energy of the initial state, calculated at 1200 K. This temperature was chosen due to the slight endothermic character of methane decomposition over carbonaceous surfaces, in which the process is generally conducted at temperatures between 800 °C and 1,000 °C (Xie et al., 2018; ? Chang et al., 2021; Fan et al., 2021; Pinilla et al., 2008).

**FIGURE 1:**
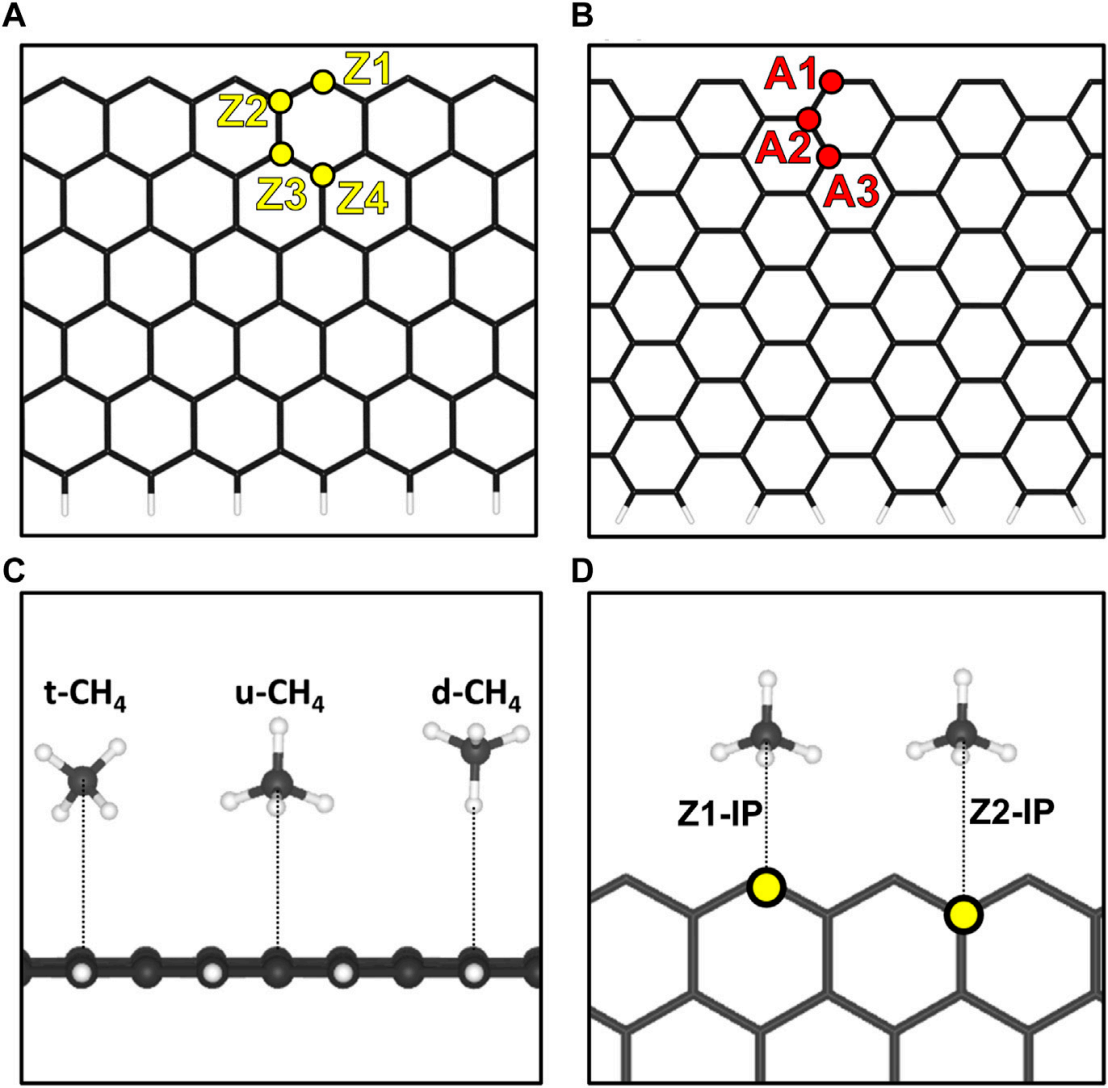
Representation of the **(A)** 12-ZGNR model and the unique-symmetry adsorption sites, Z1, Z2, Z3 and Z4 and the **(B)** 12-AGNR model and the unique-symmetry adsorption sites A1, A2, A3 and A4, investigated in this work. To better illustrate, the upper edge are hydrogen free whereas the lower edge is passivated with hydrogen. **(C)** Depictions of the orientations of methane with respect to the graphene nanoribbons, considered in this work. **(D)** Adsorptions sites for the methane in the same plane of the 12-ZGNR.

As defined before, GNRs can be classified depending on their edges, being defined as zigzag-edged GNR or armchair-edged GNR. Regarding their width, we adopted models consisting of 12 carbon atoms along the zigzag (12-ZGNR) and armchair (12-AGNR) lines between the edges of the non-periodic lattice as can be seen in Figure 1A, B, respectively. Furthermore, we constructed supercells comprising 72 carbon atoms for 12-ZGNR and 96 carbon atoms for the 12-AGNR, with a vacuum region of 20 Å in the direction perpendicular to the GNRs plane to avoid spurious interaction with adjacent periodic images. As further discussed in the following section, we adopted models consisting of hydrogen-passivated (H-terminated) and hydrogen-free (open-edge) GNRs, as catalysts for the dehydrogenation of methane. Therefore, the width of 12 carbon atom lines for the nanoribbon was adopted, since previous experimental work reported the observation of two hydrogen adatoms on the same zigzag-edge site, after the synthesis of H-terminated 12-ZGNRs (Ruffieux et al., 2016). Furthermore, a similar width of nanoribbons was adopted in previous theoretical works (He et al., 2022; Pizzochero and Kaxiras, 2022), from which adsorption energy values were suggested to be slightly altered in nanoribbons wider than 12-GNRs (He et al., 2022) Finally, previous studies showed that zigzag edges in graphene exhibit antiferromagnetic aligned edges (Son et al., 2006), therefore, spin-polarized DFT calculations were performed for 12-ZGNR.

## 3 Results

### 3.1 Physisorption of CH_4_

Pristine zigzag edges exhibit a metallic behavuour whereas pristine armchair edges behave as a semiconductor. Their band structures can be altered by atomic or molecular doping, functionalisation and adsorbed molecules (López-Urías et al., 2020; 2021; Suman et al., 2020; He et al., 2022). The band structure and total density of states (DoS) plots for the 12-ZGNR and 12-AGNR were calculated and were found to compare well with those reported in the literature as shown in Supplementary Figure S1. A uniform (10 × 1 × 1) k-point sampling grid was adopted for the band structure calculations. The metallic behaviour of the 12-ZGNR was evidenced by the crossing of conduction and valence bands on the Fermi level (Supplementary Figure S1), in agreement with previous works (Suman et al., 2020; López-Urías et al., 2021). A semiconducting character was observed for the 12-AGNR, with a calculated band gap of 0.58 eV, which is comparable to the values of 0.83 eV obtained in the work of López-Urías et al. (2020).

We began our investigations with the first step of the reaction mechanism, i.e., the physisorption of CH_4_ on the graphene nanoribbon. In summary, four unique-symmetry adsorptions sites in a surface normal, i.e., the out-of-plane (OP) direction of 12-ZGNR, were studied here and reported in Figure 1A. The adsorption sites were labelled as Z1, Z2, Z3 and Z4, with the former indicating the carbon atom on the edge itself. Adopting the same pattern, three unique-symmetry adsorption sites, in the OP direction, were studied for the 12-AGNR: A1, A3 and A3 (Figure 1B). Furthermore, we considered the physisorption of CH_4_ in the in-plane (IP) direction of the graphene nanoribbon, which was labelled Z1-IP and Z2-IP, with respect to 12-ZGNR (Figure 1D), and A1-IP and A2-IP for the 12-AGNR. For the investigation of the CH_4_ physisorption, we adopted the 12-ZGNR fully passivated with hydrogen atoms (Barone et al., 2006).

We performed a series of electronic energy calculations, with methane initially positioned at 6 Å from the Z1 site (12-ZGNR) and decreasing the distance by 0.25 Å until a distance of 2 Å from the adsorption site was reached. It is noteworthy that the distances between CH_4_ and the GNR were measured between the atom of methane positioned the closest to the GNR surface, for each CH_4_ conformation. We considered three possible orientations of methane for the construction of the potential energy surface: methane with one hydrogen atom oriented in the opposite direction from the surface (u-CH_4_—Figure 1C), methane with one hydrogen atom pointing towards the surface, in a perpendicular orientation (d-CH_4_—Figure 1C) and CH_4_ in a tilted orientation in respect to the surface (t-CH_4_—Figure 1C). Weak dispersion forces due to long-range electron correlation are expected to be the predominant interaction in the physisorption of methane (Sacchi et al., 2011; Sacchi et al., 2012a; Sacchi et al., 2012b). Therefore, we constructed potential energy surface curves for the d-CH_4_, u-CH_4_, t-CH_4_ conformations, adopting the TS and MBD dispersion corrections. Curves are reported in Supplementary Figure S2.

**FIGURE 2:**
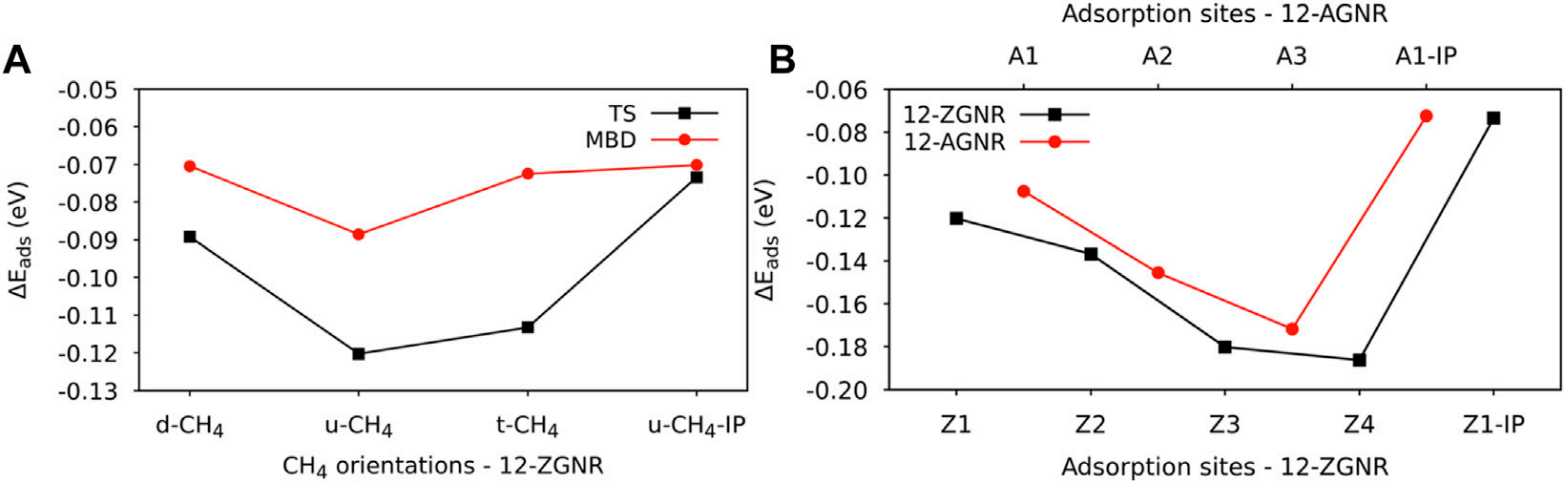
**(A)** Adsorption energy values for the CH_4_ orientations on the Z1 adsorption site of the 12-ZGNR, adopting the TS and MBD dispersion correction. **(B)** Adsorption energy values for the most favourable adsorption orientation of methane (u-CH_4_) on the adsorption sites of 12-ZGNR and 12-AGNR, investigated here.

The distances between each methane orientation and the surface, at the minimum energy configuration of the constructed PES, were adopted as starting points for geometry optimizations on the Z1 and Z1-IP adsorption sites of 12-ZGNRs. Adsorption energy values, adopting the TS and MBD dispersion corrections, were obtained as described in Eq. 1 and the results reported in Figure 2A. Overall, the u-CH_4_ configuration was the most favourable physisorption configuration on the out-of-plane Z1 site, with adsorption energy values of −0.120 eV and −0.088 eV, obtained by adoption of the TS and MBD corrections, respectively. Only the u-CH_4_ conformation was considered in investigations with respect to the Z1-IP sites, since it is the most favorable conformation in the OP adsorption sites. Adsorption energies were determined as −0.073 eV and −0.070 eV, adopting the TS and MBD corrections, respectively. Equilibrium distances between u-CH_4_ and the carbon from the Z1 site were of 3.51 Å and 3.79 Å, obtained with the TS and MBD schemes, respectively.

With respect to the different dispersion corrections considered in this study, the MBD scheme yielded the same adsorption stability order of methane on the Z1 site of the 12-ZGNR as the TS correction, although we observed a divergence in the equilibrium distance between the methane and the out-of-plane adsorption site, as shown in Supplementary Table S1. The largest deviation for the adsorption energy was obtained for the t-CH_4_, from which PBE-TS calculations predicted a value 41 meV more negative, in comparison with PBE-MBD. The equilibrium physisorption distance values, obtained from MBD corrections, were 0.21 Å, 0.28 Å and 0.34 Å higher than values obtained from at the PBE-TS level, for the d-CH_4_, u-CH_4_ and t-CH_4_ conformations, respectively. A negligible difference in the equilibrium distance was obtained when comparing the physisorption of u-CH_4_ on the Z1-IP site, from which both methods resulted in roughly 2.80 Å. Therefore, due to the higher computational cost of the MBD methodology, resulting in similar results obtained when adopting the TS dispersion correction, the latter was adopted in further calculations.

Further investigations were made for the d-CH_4_, u-CH_4_ and t-CH_4_ physisorbed conformations on the Z2, Z3 and Z4 adsorption sites. The adsorption energy values for the methane on the u-CH_4_ and t-CH_4_ orientations remained similar among all adsorption sites, varying between 4 meV and 7 meV. The u-CH_4_ was the most stable orientation on the Z1, Z3 and Z4 sites by 7 meV, 3 meV and 6 meV, respectively, whereas t-CH_4_ was the more stable at the Z2 site by 4 meV, as can be seen in Supplementary Table S2. Due to the u-CH_4_ being the most stable physisorbed conformation on the majority of the adsorption sites studied here, on 12-ZGNR, it was adopted as a reference for comparison of the adsorption energies among the Z1, Z2, Z3, Z4 and Z1-IP sites, on the 12-ZGNR, and among A1, A2, A3 and A1-IP sites on the 12-AGNR. As shown in Figure 2B, the physisorption of methane was more favourable on the Z4 (12-ZGNR) and A3 (12-AGNR) sites, with *E*_*ads*_ values of −0.186 eV and −0.171 eV, respectively and equilibrium distances of 3.38 Å and 3.47 Å, respectively. In fact, the CH_4_ physisorption energy on non-edge sites rapidly approached the adsorption energy value of methane on pristine graphene as the distance between the site and the edge grows, as evidenced by the comparison of our results with literature reports (Wood et al., 2012; Anithaa et al., 2017; Vekeman et al., 2018). In the work of Anithaa et al. (2017), the adsorption energy obtained for the physisorption of methane in the middle of a pristine graphene layer was of −0.183 eV. Wood et al. (2012) reported values of −0.175 eV for the physisorption of methane at the middle of graphene, obtained from van der Waals corrected DFT. We perform calculations for methane physisorbed at the middle of the 12-ZGNR and 12-AGNR, and we obtained values of −0.183 eV and −0.187 eV, which are similar to the adsorption energy values for the physisorption of CH_4_ on the sites Z4 and A3 (−0.180 eV and −0.186 eV, respectively). Therefore, it is possible to assume that methane can also be physisorbed on the edges under the reaction conditions of CMD, due to the physisorption of CH_4_ in the middle of graphene being only 0.08 eV and 0.06 eV more stable than the adsorption on the Z1 and A1 sites, respectively, which are lower than the thermal energy at 1200 K (0.1 eV).

**FIGURE 3:**
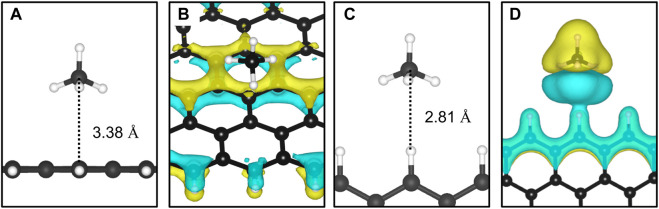
The most favourable physisorption mode of CH_4_ in respect to the **(A)** out-of-plane 12-ZGNR (Z4 site) and **(C)** in-plane 12-ZGNR. Charge density difference plots for each respective adsorption site are shown in **(B)** and **(D)**. Charge depletion is represented by blue isosurfaces and positive charge accumulation is depicted in yellow isosurfaces. The adopted isosurface cut-off value was 0.04 e Å^−3^.

The adsorption of methane on the out-of-plane adsorption sites were at least 46 meV more stable than the in-plane Z1-IP adsorption site, from which an *E*_*ads*_ value of −0.070 eV was obtained, in excellent agreement with previous DFT results (Wood et al., 2012). However, when adopting the same computational methodology, Wood et al. (2012) reported that no stable minimum was found for out-of-plane adsorption sites of 12-ZGNR. The adsorption energy of methane over the in-plane sites of 12-ZGNR and 12-AGNR was roughly the same, diverging by about 1 meV, which is likely due the zigzag and armchair edges being passivated with hydrogen. Depiction of the most favourable physisorption configurations of CH_4_ on the Z4 (Figure 3A) and Z1-IP (Figure 3C) sites, as well as their equilibrium distances, were reported in Figure 2A, C, respectively. Chemical insights were gained about the preference for physisorption of methane on OP sites of graphene nanoribbons, in comparison with the IP site, by plotting the charge density difference between the adsorbed system, isolated molecule and GNR. Figure 3 shows the positive charge accumulation (yellow region) and charge depletion (blue region) of the methane physisorption on Z4 and Z1-IP sites of 12-ZGNR. The lower stability of the IP site can be attributed to the presence of a stronger repulsive electrostatic component in the bonding, suggested by the charge accumulation from the in-plane methane physisorbed mode and charge depletion on the edges, showed in Figure 3D. It can be seen in Figure 3B that the surface is polarised with positive charge density in the 12-ZGNR backbone whereas a stronger charge depletion region is observed in the edge sites. Therefore, we can infer that more intense dispersion interactions are present between methane and the Z4 physisorption site.

### 3.2 First deprotonation of CH_4_

The first deprotonation step of CMD (CH_4_ → CH_3_ + H) was investigated for two possible mechanisms: the direct (dissociative) and precursor-mediated (non-dissociative) methane adsorption (Alves et al., 2021; Fan et al., 2021; Wang et al., 2021; Pan et al., 2022). For the former, CH_3_ and H are expected to chemisorb at the surface of the catalyst while the first C-H bond of methane cleavage occurs simultaneously. For the precursor-mediated mechanism, the chemisorption and the C-H dissociation occurs in separate steps. The mechanism of methane adsorption over a solid catalyst is crucial for identifying the rate-limiting step of the catalytic methane decomposition (Hamdan et al., 2022; Tong et al., 2022). Therefore, a detailed investigation is presented in this section. Hereafter, the active site on the edge in which the species is chemisorbed is defined by “/Z1″, for the 12-ZGNR and “/A1” for the 12-AGNR.

### 3.3 Dehydrogenation on H-terminated 12-ZGNR and 12-AGNR edges

To investigate the first reaction mechanism step of the catalytic dehydrogenation of CH_4_, forming CH_3_ and H on the 12-ZGNR and 12-AGNR edges, it is necessary to elucidate the most favourable chemisorption sites of CH_3_ and H among the sites detailed in Figure 1 and correlate with the methane adsorption on edges. Adsorption energy values on the hydrogen-passivated edges of 12-ZGNR and 12-AGNR range from −2.463 eV (Z1) to −0.251 eV (Z2) for the methyl chemisorption on the 12-ZGNR. Adsorption energy values between −1.421 eV (A1) and −0.277 eV (A3) were observed for the CH_3_ chemisorption on 12-AGNR (see Table 1). We investigated the adsorption of CH_4_ into CH_3_* and H* (the star index indicates chemisorbed species) at 1200 K on the hydrogen-terminated Z1 and A1 sites (Supplementary Figure S3) obtaining a free energy of activation of 3.76 eV and 3.95 eV, respectively. The high Δ*G*_*a*_ values for methane adsorption are likely due to deactivation by the strong chemisorption of hydrogen atoms on the passivated edges, evidenced by the *E*_*ads*_ value of −2.830 eV and −1.421 eV (Table 1) on the Z1 and A1 sites respectively, in agreement with previous theoretical results (Pizzochero and Kaxiras, 2022). Finally, the investigation of the dehydrogenation on the 12-ZGNR and 12-AGNR passivated edges is necessary to evaluate the availability of the bare carbon active site and the viability of the CMD process.

**TABLE 1:** Chemisorption energy (eV) for the CH_3_ and H, calculated for the Z1, Z2, Z3 and Z4 sites of 12-ZGNR and for the A1, A2 and A3 sites of 12-AGNR.

12-ZGNR			12-AGNR		
	$E_{ads,{\text{CH}}_{3}}$ (eV)	*E*_*ads*,H_ (eV)		$E_{ads,{\text{CH}}_{3}}$ (eV)	*E*_*ads*,H_ (eV)
Z1	−2.463	−2.830	A1	−1.010	−1.421
Z2	−0.251	−0.447	A2	−0.587	−0.931
Z3	−1.284	−1.622	A3	−0.277	−0.613
Z4	−0.561	−0.885			

**FIGURE 4:**
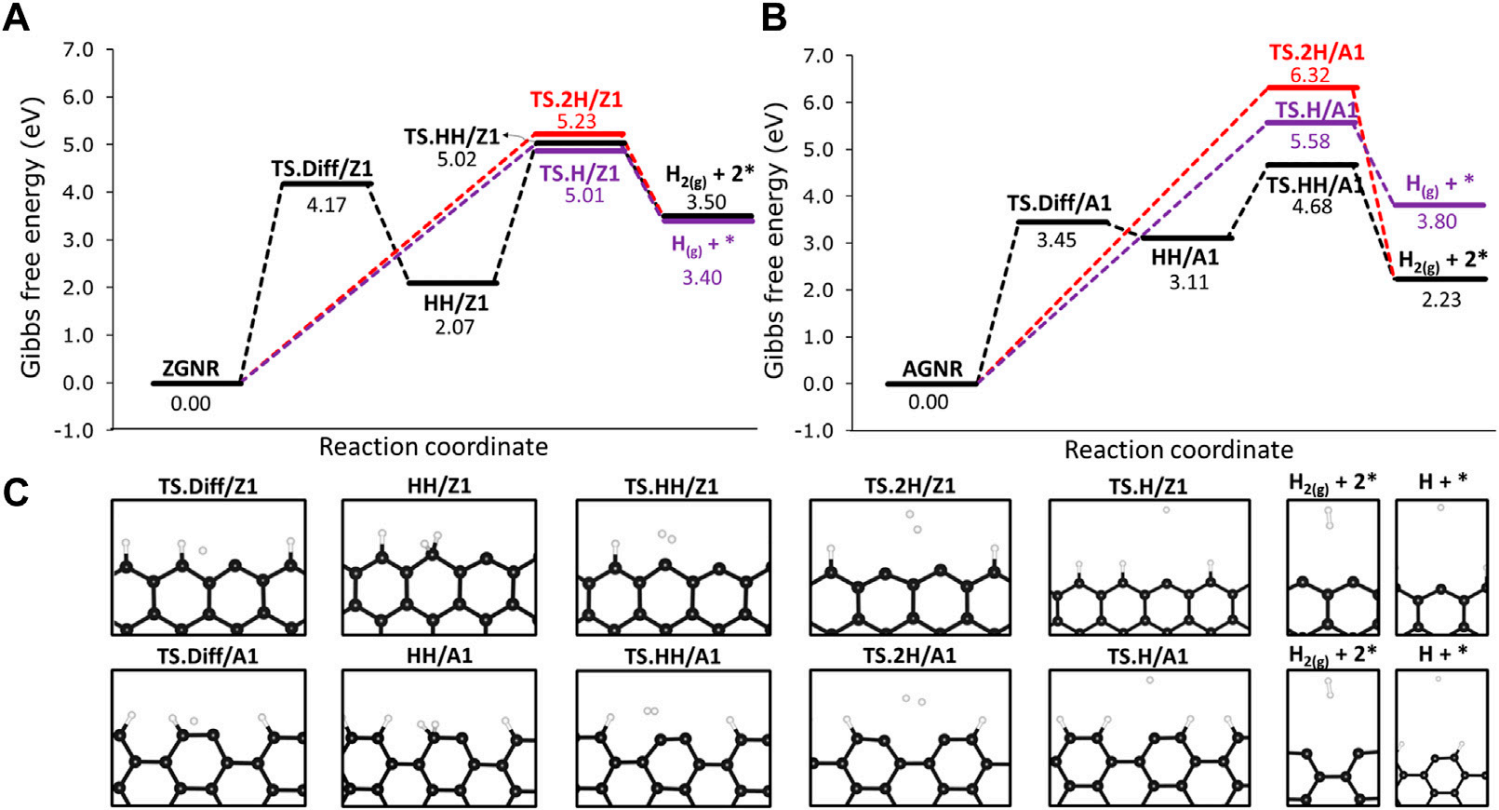
Free energy reaction profile, at 1200 K, of the dehydrogenation on H-passivated **(A)** zigzag edges and **(B)** armchair edges. **(C)** Depiction of the stationary point located in the reaction mechanisms and their respective labels. Reaction coordinate is in arbitrary units.

We investigated the H_2_ formation reactions from the passivated Z1 and A1 sites by analysing the combination of hydrogen atoms chemisorbed on the edges. For the former, we labelled as 2H/Z1 and 2H/A1 when the hydrogen atoms are chemisorbed on different edge sites, as shown in Figure 4C. Following the same approach, we labelled as HH/Z1 and HH/A1 when both hydrogen atoms are chemisorbed on the same edge site (Figure 4C). We also made calculations for hydrogen diffusion on 12-ZGNR and 12-AGNR edges. Furthermore, we analyse the hydrogen atom desorption from the graphene edges and the results are reported in Figure 4. Overall, the combination of chemisorbed hydrogen on different sites into H_2_ have activation free energy of 5.23 eV and 6.32 eV, respectively. Similar results are obtained for the atomic hydrogen desorption on 12-ZGNR and AGRN due to high Δ*G*_*a*_ values of 5.01 eV and 5.58 eV. The most feasible pathway for a bare carbon active site formation is from the recombination of the hydrogen adatom from the edges (HH/Z1 and HH/A1) forming H_2_. On the 12-ZGNR, the HH/Z_1_ → TS ⋅HH/Z_1_ → H_2_ reaction proceeds through a barrier of 2.92 eV, whereas a Δ*G*_*a*_ of only 1.56 eV is necessary on the 12-AGNR. Hydrogen diffusion on the 12-ZGNR and AGNR passivated edges can occur by forming an hydrogen adatom with a Δ*G*_*a*_ of 4.17 eV and 3.45 eV, respectively, being the rate determinant step for the H_2_ desorption from HH/Z1 and HH/A1. It is noteworthy that the barriers of methane adsorption on passivated edges have free energy of activation values 0.41 eV lower on 12-ZGNR and 0.50 eV higher on 12-ZGNR and 12-AGNR, respectively, being possible to occur at higher temperatures in which the process is conducted. However, further dehydrogenation steps of methane on passivated edges are unlikely to occur to the deactivation by hydrogen atoms and this scenario was not considered in the following investigations.

### 3.4 Methane deprotonation on H-free (open edge) 12-ZGNR and 12-AGNR

As it was stated in the last section, the availability of unpassivated carbon active sites is likely at the high temperatures in which the CMD process is conducted, with the H_2_ desorption from armchair edges being the most kinetically feasible, therefore, we have investigated the CMD steps on the bare carbon active sites of the edges of 12-ZGNR and 12-AGNR, i.e., adopting a fully dehydrogenated edge (open edge). Experimental evidence confirms that hydrogen-free graphene edges are expected to exist even in vacuum conditions (He et al., 2014).). In fact, hydrogen-free edges have been adopted for the investigations of the growth of epitaxial graphene (Wu et al., 2019), and the reconstruction of the bare graphene zigzag and armchair edges (Gao et al., 2012; Li et al., 2013; Soldano et al., 2014). Moreover, it is expected that the non-hydrogenated edge reactive sites exist at high temperatures, at which the CMD process takes place (Calderón et al., 2016). A similar model, adopting dangling carbon atoms on edge, was adopted for the investigation of methane formation (Calderón et al., 2016) and CO_2_ adsorption (Montoya et al., 2003; Noei, 2016) on edges of carbonaceous surfaces, with good agreement with experimental data (Montoya et al., 2003). For completeness, we also investigated the stability of the H-passivated and H-free ZGNR and AGNR, by performing *ab initio* molecular dynamics (AIMD) simulations at 1200 K and results are presented in Supplementary Figure S4 of the Supplementary Material. After 1 ps simulations, all the structures remained stable and no deformation or Stone-Wales transformation were observed, showing that the catalyst model is appropriate for further investigations of the deprotonation of methane. We added in Supplementary Figure S5 a snapshot of the AIMD simulations at different time steps of the simulation, and we observed a equilibrium distance between 6.04 Å and 6.81 Å obtained at 500 fs and 1,000 fs, respectively.

**FIGURE 5:**
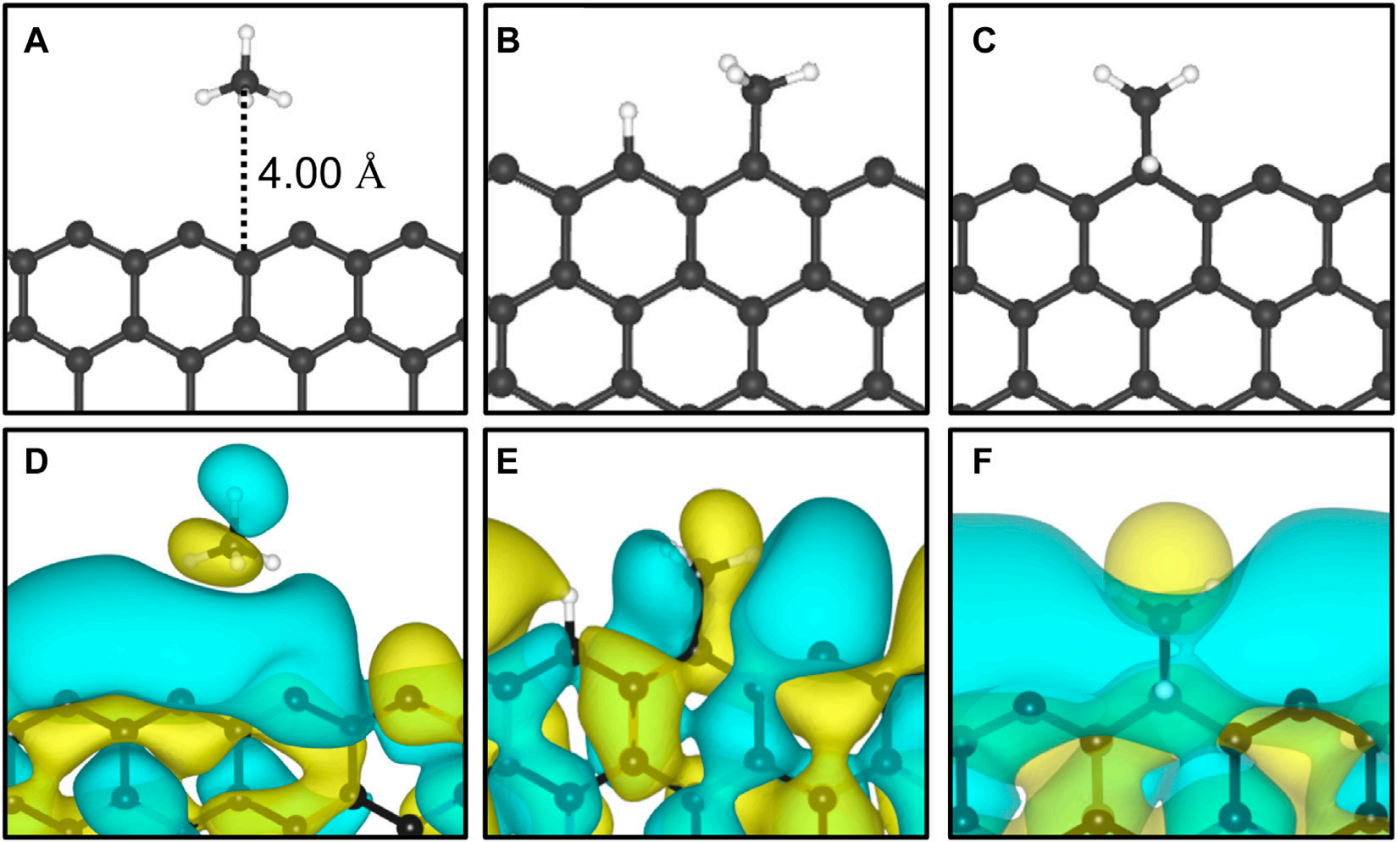
Representation of **(A)** CH_4_ adsorbed on the Z2; **(B)** CH_3_ and H chemisorbed on different Z1 sites; **(C)** CH_3_ and H chemisorbed on the same Z1 site and their HOMO represented in **(D, E)** and **(F)**, respectively. The yellow and cyan colors represent the positive and negative phases, respectively, of the HOMO. The adopted isosurface cut-off value was 0.01 e Å ^−3^.

Two possible pathways for methane deprotonation were proposed on the 12-ZGNR and 12-AGNR edges: the first reaction pathway leads to the production of CH_3_ and H chemisorbed on different Z1 (12-ZGNR) or A1 (12-AGNR) adsorption sites, while the second pathway leads to CH_3_ and H chemisorbed on the same Z1 (or A1) site, as depicted in Figure 5B, C, respectively. The chemisorption of the methyl moiety and the hydrogen atom on the edges occurs in the plane of the nanoribbon (Figure 5). In this regard, the physisorption of CH_4_ on the Z2 site (bridge site—Figure 1) is more favourable than the adsorption on Z1 by only 4 meV. Similarly, we observe that the physisorption of CH_4_ on the A2 site of the 12-AGNR is energetically more favourable than on the A1 site by 21 meV. Depiction of the physisorption structure over the Z2 site is shown in Figure 5A.

**FIGURE 6:**
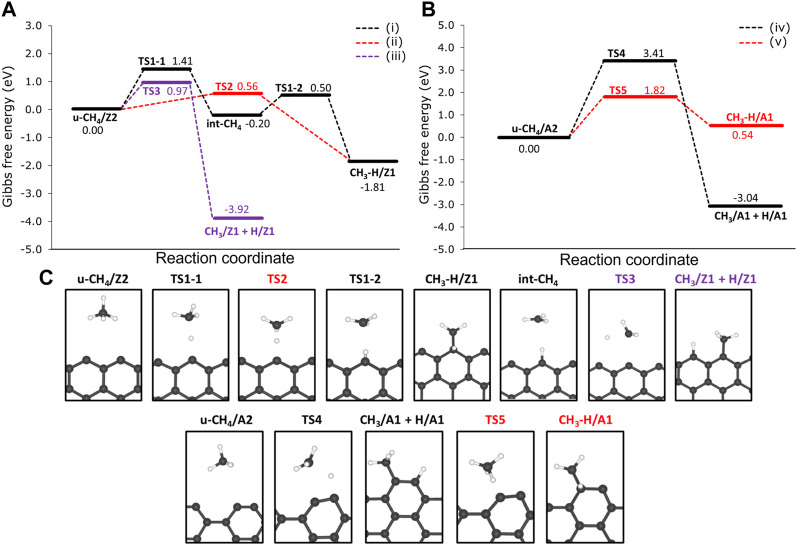
Free energy reaction profiles at 1200 K for the dehydrogenation of methane on the non-passivated edges of **(A)** 12-ZGNR and the **(B)** 12-AGNR. The zero energy is relative to the most stable physisorbed methane conformation on the 12-ZGNR and 12-AGNR edges. In **(A)**, the black line refers to the mechanism i), which is the precursor-mediated mechanism of CH_4_ adsorption into CH_3_ and H, onto 12-ZGNR edges; The red and purple lines represent the direct mechanism ii) of CH_4_ deprotonation on the same Z1 site and different Z1 sites iii) of 12-ZGNR edges, respectively. In **(B)**, black lines and red lines represent the direct mechanisms of methane adsorption on different A1 sites and on the same A1 sites of 12-AGNR edges, respectively. Reaction coordinate is in arbitrary units. The stationary points of the reaction mechanism and their labels are depicted in **(C)**.

We investigated two reaction pathways connecting the physisorbed CH_4_ to the CH_3_ and H chemisorbed on the same Z1 edges via: 1) a two-step mechanism, from which hydrogen is chemisorbed on Z1 in the first step, followed by the chemisorption of CH_3_ on the same Z1 site (precursor-mediated mechanism) and a 2) one-step mechanism from which CH_3_ and H are chemisorbed in the same step (direct dissociative mechanism). Reaction profiles of the 1) and 2) mechanisms are presented in Figure 6A, as well as labels for each of the stationary points of the reaction mechanism. The reaction mechanism 1) consists of two parts: firstly, the migration of H from CH_4_ to the Z1 site through a free energy barrier of 1.41 eV (TS1-1). Here the methyl radical is stabilized by the hydrogen atom migrated to the edge of the int-CH_4_ structure. Secondly, the TS1-2 connects int-CH_4_ to the CH_3_ and H chemisorbed onto the same Z1 site (hereafter referred to as CH_3_-H/Z1) through a Δ*G*_*a*_ of 0.50 eV.

Our study suggests that the precursor-mediated i) and direct ii) mechanisms of methane deprotonation occur competitively (Figure 6A). For the latter, a one-step mechanism was proposed, passing through the transition state TS2 and forming CH_3_–H/Z1 with an activation free energy of activation of 0.56 eV. Due to the lower Δ*G*_*a*_ value, it is expected that mechanism ii) is preferred over i) and the C-H bond cleavage is followed by the symmetric chemisorption of methyl and hydrogen. We also investigated the chemisorption of the methyl moiety and the hydrogen atom in different Z1 sites iii), as shown in Figure 6A. In this pathway, we found that the chemisorption of CH_3_ and H proceeds synchronously, with u-CH_4_/Z2 reacting through TS3, with a Δ*G*_*a*_ of 0.97 eV.

The reaction mechanism ii) is the more favourable reaction pathway, with a Δ*G*_*a*_ 0.41 eV lower than iii) and 0.85 eV lower than the rate-determining step (migration of the hydrogen atom to the edge) of mechanism i). Therefore, a thermodynamically controlled reaction is possible, from which the reaction pathway iii) is expected to be more relevant at higher temperatures. This possibility is suggested by the very exothermic character of the dehydrogenation reactions of methane on the graphene nanoribbon edges (Δ*G* = −3.92 on 12-ZGNR edges and Δ*G* = −3.04 on 12-AGNR edges), as presented in Figure 6. A better picture of the thermodynamic control of reaction pathway iii) can be seen in Supplementary Figure S6, from which the concentration of the thermodynamic product, CH_3_/Z1-IP + H/ZI-IP was obtained as a function of time, at different temperatures. As expected, the formation rate of the product CH_3_/Z1-IP + H/ZI-IP is heavily influenced by the temperature and the complete conversion was achieved, roughly 130 times faster at 900 K, in comparison with conversion time at 800 K and 60 times faster at 1000 K, in comparison with the conversion time at 900 K. Further insights were obtained by inspection of the HOMO orbitals of both products, CH_3_/Z1 + H/Z1 and CH_3_-H/Z1, as reported in Figure 5. It is possible to observe a greater overlap between the orbitals of the methyl and hydrogen fragments on different sites (CH_3_/Z1 + H/Z1) in Figure 5E, in comparison with the HOMO orbitals in CH_3_-H/Z1 (Figure 5F). For the latter, the *π* system formed by the dangling bonds of the GNR edge are the most dominant in the adsorbed system, contributing to its lower stability in comparison with CH_3_/Z1 + H/Z1. Therefore, the interactions between the products and the dangling bonds result in an overall better stability of the CH_3_ and H chemisorbed on different Z1 sites, in agreement with the experimental findings of the structural stability of graphene nanoribbons (Barone et al., 2006).

The reaction mechanism for the CH_4_ dehydrogenation on the 12-AGNR is summarised in Figure 6B. Here, two competing one-step mechanisms for CH_4_ deprotonation on the 12-AGNR edges lead either to the synchronous chemisorption of CH_3_ and H on adjacent A1 sites (mechanism iv) or to the chemisorption of the products on the same A1 site (mechanism v). It is expected that the adsorption of methane proceeds through TS4 iv), with an energy barrier height of 3.41 eV and forming CH_3_/A1 + H/A1. We note that the carbon atom of the A1 site slightly relaxes towards the direction of the nanoribbon, as can be seen in Figure 6B. This occurs to minimise the steric hindrance between the methyl and the carbon from the A1 site during chemisorption. A more kinetically favourable reaction, in comparison with iv), was suggested through the v) mechanism, which is characterised by a rate-determining-step with a barrier height of 1.82 eV for the synchronous chemisorption of CH_3_ and H on the same A1 site. As elucidated before, the synchronous chemisorption of CH_3_ and H on different adsorption sites on the edge is expected to provide more thermodynamically stable products. Therefore, the formation of CH_3_/A1 and H/A1 products is exothermic, being 2.50 eV more stable than the chemisorption of the same moieties on the same adsorption site.

From the results presented in this section, we conclude that the first step of methane decomposition on the edges of graphene nanoribbons proceeds through a direct dissociation pathway and occurs more favourably on GNRs with zigzag edges. The discussion presented in this paragraph is based on the DFT energy values obtained here, detailed in Table 2, and reported in the literature. The dissociative chemisorption mechanism of CH_4_ presented here, proceeds through a barrier of 0.45 eV (Δ*G*_*a*,1200*K*_ = 0.56 eV), which is significantly lower than what is reported (in the 0.54 eV–1.80 eV range) for the decomposition of methane over transition metal surfaces such as Ni, Fe and Ru (KOERTS, 1992; Salam and Abdullah, 2017). Several molecular beam studies combined with first-principle calculations have been reported for the direct dissociation of CH_4_ on single-crystal metal surface such as Pt (111) (Bisson et al., 2007; Guo and Jackson, 2016), Pt (110) (Sacchi et al., 2011; Bisson et al., 2010a; b) and Ni(111) (Bisson et al., 2007; Nave and Jackson, 2009). There is generally an excellent agreement between the prediction of DFT calculations and the experimental barrier heights, 1.1 eV for Ni(111) (Shen et al., 2015) and 0.8 eV for Pt (111) (Guo and Jackson, 2016). Therefore we are confident that the most favourable calculated chemisorption barrier for methane over GNR is about 0.35–0.65 eV lower than both metal surfaces. The stretched C-H bond length at the transition state of methane dissociation into CH_3_ and H on Ni(111), Ni(100) and Pt (110) surface was predicted to be 1.63 Å, 1.66 Å and 1.67 Å, respectively Shen et al. (2015); Sacchi et al. (2011); Anghel et al. (2005). Here, we obtained a stretched C-H bond length of 1.27 Å for the most favourable transition state on 12-ZGNR, meaning that the dissociation of CH_4_ over GNRs has a much “earlier” barrier, using a Polanyi framework (Ebrahimi et al., 2010), than over Pt and Ni metal surfaces. The lower energy calculated for the methane adsorption on 12-ZGNR edges is likely due to the C-H bond length from the transition state being closer to the equilibrium C-H bond length of gas-phase methane of 1.09 Å, in comparison with the transition state of methane adsorption on Ni (111). Due to the consistency between experimental and computational results for the chemisorption of methane, we are confident in our proposed mechanisms. The barrier for methane chemisorption on GNR is lower than for Ni and Pt by 0.35–0.65, which may have catalytic implications.

**TABLE 2:** The DFT energy barrier heights and activation free energy values at 1200 K (inside parenthesis) obtained here and compared with theoretical values and experimental apparent activation energy available in the literature, in eV, for the reaction steps of CMD mechanism (S1: CH_4_→ CH_3_*+ H*; S2: CH_3_*→ CH_2_*+ H*; S3: CH_2_*→ CH*+ H*; and S4: CH*→ C*+ H*. The catalyst adopted in the respective work is indicated.

Catalyst	S1	S2	S3	S4
12-ZGNR edges (this work)	0.45 (0.56)	1.16 (1.24)	1.57 (1.53)	1.1 (1.06)
Ni(111) (Li et al., 2014)	1.23	0.85	0.29	1.36
ZGNR edges w/vacancy^a^ (Calderón et al., 2016)	2.82	1.69	3.322^b^	0.45
Ni-*γ*Al_2_O_3_ (Salam and Abdullah, 2017)	0.98	0.63	1.15	0.63
Pd-*γ*Al_2_O_3_ (Salam and Abdullah, 2017)	0.003	0.34	0.33	0.21
Mo-*γ*Al_2_O_3_ (Salam and Abdullah, 2017)	0.048	3.82	1.99	5.98
Stepped-Ru (0001) (Arevalo et al., 2017)	1.02	-	-	1.10
Cu-Bi (Palmer et al., 2019)	2.80	-	-	-

^a^ Reaction steps were retrieved from the inverse reactions of the methane formation mechanism.

^b^ Occurs concomitant with H_2_ formation.

### 3.5 Further steps in the dehydrogenation of CH_4_ on the 12-ZGNR edges

**FIGURE 7:**
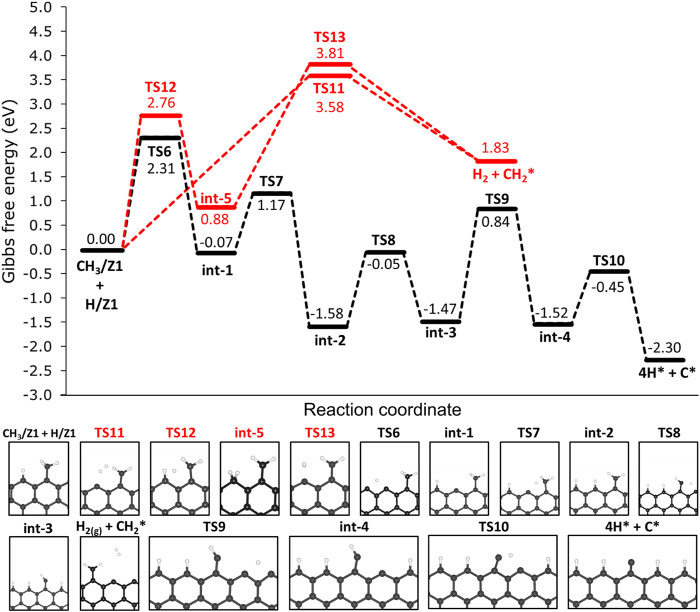
Free energy profiles for the consecutive dehydrogenation of CH_3_/Z1 + H/Z1 on the non-passivated edges of 12-ZGNR (upper panel). Lower panel: depiction of the stationary points presented in the reaction profile Gibbs free energy values are relative to methyl and hydrogen chemisorbed on Z1 sites. Reaction coordinate is in arbitrary units.

As elucidated before, the most thermodynamically favourable products of the deprotonation of methane are CH_3_/Z1 and H/Z1. Consecutive deprotonation steps were investigated and the reaction profile is presented in Figure 7. Hereafter, the species chemisorbed on the edges are being represented by a star index ‘*’, since we are referring to the same active site on the 12-ZGNR (Z1) and on 12-AGNR (A1). In the first step, the chemisorbed hydrogen on the edge (H/Z1) diffuses to the next Z1, passing through TS6 with an activation free energy of 2.31 eV, and forming int-1 CH_3_/Z1 + H/Z1 → TS7 → int-1. Following the diffusion of H, the cleavage of the C-H bond from CH_2_ is expected to occur through TS7 (int-1 → TS7 → int-2 with a Δ*G*_*a*_ value of 1.24 eV. The consecutive steps of hydrogen diffusion and CH_2_ deprotonation, occur from int-2 and pass through two consecutive transition states: int-2 → TS8 → int-3 and int-3 → TS9 → int-4 with free energy barriers of 1.63 eV and 2.31 eV, respectively. An activation free energy of 1.07 eV was calculated for the last step of the reaction mechanism (int-4 → TS10 → 4H* + C*), resulting in a carbon atom and four hydrogen atoms chemisorbed on the edges. The final dehydrogenation products on the 12-ZGNR edges (4H* + C*) are predicted to be 2.30 eV more stable than the initial chemisorbed CH_3_/Z1 and H/Z1. Overall, the free energy barriers of the diffusion reactions were higher than deprotonation barriers by an average of 1.03 eV, therefore, the diffusion of H is the rate-determining step of the dehydrogenation mechanism of methane on 12-ZGNR edges.

The mechanisms for H_2_ formation were investigated, and three possible pathways were obtained. From CH_3_/Z1 + H/Z1, H_2_ is expected to be formed through the transition state TS11 (CH_3_/Z1 + H/Z1 → TS11 → H_2_ + CH_2_*). In this pathway, one hydrogen atom from the chemisorbed CH_3_/Z1 and the hydrogen atom chemisorbed on the edge reacted forming H_2_, with a Δ*G*_*a*_ of 3.58 eV. Another possible reaction pathway was obtained from CH_3_/Z1 + H/Z1, in which a s C-H bond cleavage occurs and the hydrogen atom migrates to the edge site already occupied with a proton (CH_3_/Z1 + H/Z1 → TS12 → int-5). This process proceeds through TS12, with a Δ*G*_*a*_ of 2.76 eV. The H_2_ is formed from int-5, from the two hydrogen atoms chemisorbed on the same edge, with a barrier height of 2.93 eV. Based on the results reported in Section 3.2.1, the most likely scenario of the bare carbon active sites regeneration under reaction conditions, i.e., the desorption of H_2_ from the edges, is through the diffusion of H, forming HH/Z1 (12-ZGNR → TS. DIff/Z1 → HH/Z1, Δ*G*_*a*_ = 4.17 eV) followed by the H_2_ formation (HH/Z1 → TS. HH/Z1 → H_2_ + *, Δ*G*_*a*_ = 2.95 eV) leaving only solid carbon on the edges which remains as an active site for further methane dehydrogenation reactions.

**FIGURE 8:**
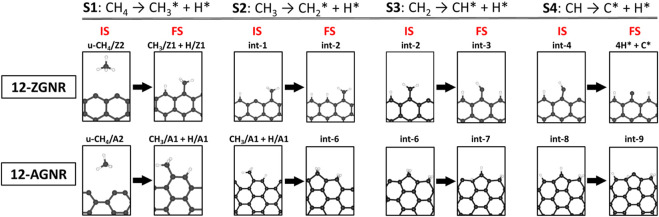
Schematic of geometries and their respective labels adopted in this work for the initial states (IS) and final states (FS) of the four steps of the CMD steps on 12-ZGNR and 12-AGNR. S1, S2, S3 and S4 stand for steps 1-4 of the CMD process.

The barrier heights (at 0 K) and activation free energy values at 1200 K of the reaction pathway proposed here, and a comparison with literature reports for the reaction steps S1 (dissociative: CH_4_ → CH_3_* + H*), S2 (CH_3_* → CH_2_* + H*), S3 (CH_2_* → CH* + H*) and S4 (CH* → C* + H*), are presented in Table 2. We have shown in Figure 8 a schematic of the initial states (IS) and final states (FS) of each CMD step (S1—S4) and the respective labels adopted in this work, aiming to facilitate the comparison with the literature data presented in Table 2. Li et al. (2014) investigated the decomposition of methane on a Ni(111) clean surface, utilizing DFT coupled with STO-3G basis set. The authors reported that methane adsorption proceeds through a dissociative mechanism (S1) with a barrier height of 1.23 eV and suggested barrier heights of 0.85 eV, 0.29 eV and 1.36 eV for the consecutive dehydrogenation reactions (S2, S3 and S4, respectively). In the work of Calderón et al. (2016), a mechanism for methane formation on the zigzag edges was proposed in an aromatic cluster consisting of five benzene rings with two edges dangling carbon atoms as reactive sites. Calculations were made at the B3LYP/6–311++G (d,*p*) level and activation energies were proposed for the temperature range between 298 K and 1500 K. The processes relevant to CMD were taken as the reversible reaction steps of the proposed mechanism and reported in Table 2. Based on their reports, the dissociative mechanism of CH_4_ adsorption (S1) occurred on a carbon vacancy on the zigzag edge, with a barrier height of 2.82 eV, whereas the H_2_ formation was suggested to occur concomitant to step S2 (see Table 2) of CMD with an energetic barrier of 3.32 eV.

In the work of Salam and Abdullah (2017), the CMD mechanism was studied for Ni, Pd and Mo-promoted *γ*-alumina, from which methane was suggested to decompose through a direct mechanism. The barrier for the most kinetically-favourable dissociation mechanism obtained here (S1, 0.45 eV), was lower than barrier height values reported for the Ni-*γ*Al_2_O_3_ (0.98 eV). When adopting other transition metals (Pd and Mo) promoting the *γ*-alumina catalyst, the direct mechanism of methane (S1) was suggested to proceed with a barrier height of 0.003 eV and 0.048 eV, respectively (Salam and Abdullah, 2017). Arevalo et al. (2017) studied the dissociative adsorption of methane (S1) and the step S4 of the CMD mechanism on a stepped Ru (0001) surface, adopting the PBE-D2/PAW methodology, reporting values of 1.02 eV and 1.1 eV, respectively. In the work of Palmer et al. (2019), the mechanism of methane decomposition was investigated on a Cu-Bi alloy catalyst by AIMD simulations. Activation energy of 2.8 eV was obtained for the dissociative mechanism (S1). The results were in good agreement with the experimental measured apparent activation energy of 2.3 eV, in the temperature range of 1123 K and 1253 K for the same reaction step.

Overall we found that the reactions occurring at the 12-ZGNR edges are comparable, in terms of activation energies and thermodynamic requirements, with metallic catalysts, based on the literature reports presented in Table 2. Only a few metal-based catalysts, such as the Pd-promoted and the Mo-promoted *γ*-alumina have lower CH_4_ dissociation barriers than 12-ZGNRs (0.44 eV and 0.40 eV lower, respectively). With respect to the entire CMD mechanism, the rate-determining step obtained on the 12-ZGNR edges was S3, with a barrier of 1.57 eV, which is higher than for the rate-determining step on Ni(111) (S1, 1.23 eV) (Li et al., 2014), Ni-*γ*Al_2_O_3_ (S3, 1.15) eV and Pd-*γ*Al_2_O_3_ (S2, 0.34 eV) (Salam and Abdullah, 2017).

### 3.6 Growth and active site regeneration on the 12-AGNR edges

**FIGURE 9:**
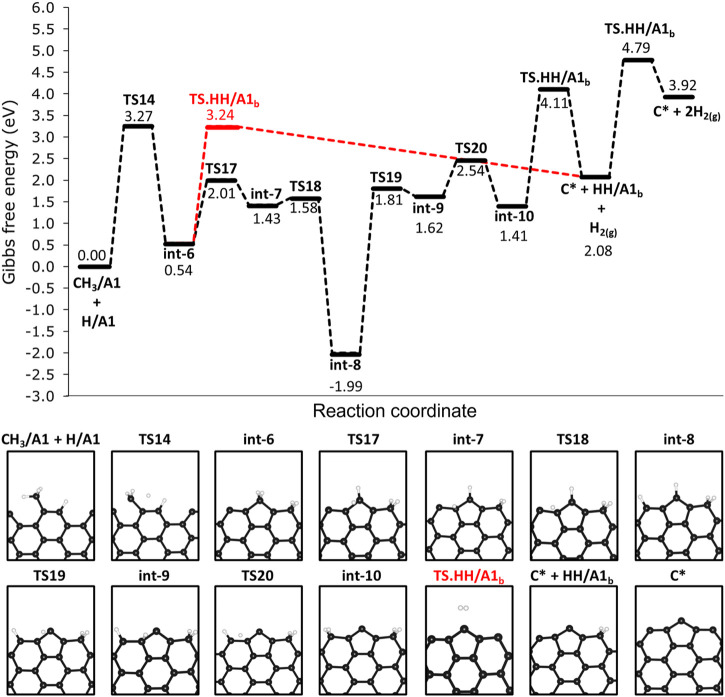
Free energy reaction profile, at 1200 K, for the deprotonation steps of methane on H-free edges of 12-AGNR (upper panel). The stationary points presented in the reaction profile are depicted in the lower panel. Gibbs free energy values are relative to methyl and hydrogen chemisorbed on A1 adsorption sites. Reaction coordinate is in arbitrary units.

We investigated the steps following the adsorption of methane on 12-AGNR edges and the results are shown in Figure 9. The deprotonation of CH_3_/A1 proceeds with an activation free energy of 3.27 eV, forming int-6, which is a label for the CH_2_/A1 and HH/A1 (CH_3_/A1 + H/A1 → TS14 → int-6). The deprotonation of the methyl is followed by the bonding of CH_2_/A1 into two rows of carbon atoms on the armchair edges, resulting in a grown active site on the edges hereafter labelled as A1_*b*_ (Figure 9). The H_2_ formation from HH/A1_*b*_ proceeds with a higher activation free energy barrier of 2.70 eV (HH/A1_*b*_ → TS. HH/A1_*b*_ → C* + H_2_) in comparison with the pristine carbon site on the armchair edge (HH/A1 → TS. HH/A1 → * + H_2_, Δ*G*_*a*_ = 1.57 eV), detailed in Figure 4B. It is noteworthy that the same activation free energy value is expected for the H_2_ formation on the carbon row containing the C* and, therefore, it is defined as the same active site and also labelled as A1_*b*_. The Δ*G*_*a*_ value of 2.70 eV is expected in the final step of the catalyst regeneration forming only a carbon atom on the surface (C* + HH/A1_*b*_ → TS. HH/A1_*b*_ → C* + H_2_) and remaining as an active site for further methane decomposition steps. An alternative pathway for the regeneration of the 12-AGNR active sites on edges proceeds from int-6 through low free energy barriers of 1.47 eV (int-6 → TS17 → int-7) and 0.15 eV (int-7 → TS18 → int-8), respective to the hydrogen atom migration among adjacent A1_*b*_ sites until reaching the last carbon on the atom row (int-8, Figure 9). The intermediate formed (int-8) is more thermodynamic stable than the previous minimum energy structures in the mechanism (int-7), by 3.42 eV. The Δ*G*_*a*_ values of 3.80 eV (int-8 → TS19 → int-9) and 0.92 eV (int-9 → TS20 → int-10) are expected for the migration of a hydrogen atom from the grown carbon site to forming the HH/A1_*b*_ (int-10, Figure 9). Finally, a consecutive reaction of H_2_ formation is expected to occur from int-10 with Δ*G*_*a*_ of 2.70 eV (HH/A1_*b*_ → TS. HH/A1_*b*_ → C* + H_2_), regenerating the A1_*b*_ active sites.

## 4 Conclusion

Much effort has been made to design an efficient and environment-friendly catalyst for producing hydrogen from methane, and nanocarbons have been proposed as one of the most suited materials for this reaction. In this work, we presented a detailed reaction mechanism, obtained through first-principles DFT calculations, for the process of methane catalytic decomposition on the edges of graphene nanoribbons. The dehydrogenation of the zigzag and armchair edges was investigated and we concluded that the bare carbon active site is mainly available due to H_2_ desorption from two hydrogen atoms chemisorbed on the sabe edge site, in which we estimated free energy activation values of 2.95 eV and 1.56 eV for the 12-AGNR and 12-ZGNR. Furthermore, we found that the direct mechanism of CH_4_ dissociation into CH_3_ and H was the preferred mechanism for catalysing the breaking of the first C-H bond of methane.

Several competing reaction mechanisms for methane dehydrogenation on 12-ZGNR and 12-AGNR were investigated. Our results show that the deprotonation of methane proceeds *via* a synchronous mechanism, in which the cleavage of the C-H bond and chemisorption of the methyl and hydrogen atom occur in the same step, i. e., direct reaction mechanism. Furthermore, the minimum reaction pathway proceeds through a small activation free energy of 0.56 eV on the 12-ZGNR edge. An alternative pathway connecting the physisorbed methane to the methyl and hydrogen chemisorbed on different sites was proposed. This reaction pathway proceeds through a barrier height of 0.97 eV and forming a more thermodynamically stable product. The highest free energy of activation among the deprotonation reactions was 1.53 eV whereas the highest Δ*G*_*a*_ value among the diffusion reactions was 2.32 eV. On the 12-AGNR edges, the deprotonation of CH_3_ generates a carbon bonded with the atom row on the armchair edges. Molecular hydrogen is desorbed from the grown active sites with an activation free energy of 2.70 eV, being easier for catalyst regeneration in comparison with the zigzag edges.

Our theoretical results compare favorably with other literature reports, providing a justification for employing pristine graphene nanoribbons as a catalyst for the CMD process since these nanocarbons show a remarkably small barrier for the dissociation of methane, comparable to the values between 0.54 eV and 1.80 eV for commonly used metal catalysts. It is noteworthy that the zigzag edges are more susceptible to deactivation from the chemisorption of hydrogen atom whereas the growth of the armchair edges acts as the regeneration of the active sites.

The results presented here provide insights for the autocatalytic activity of graphene nanoribbons, enlightening the reactivity of graphene nanoribbons edges on the methane decomposition. To the best of our knowledge, this is the first time in which the CMD reaction steps were carefully evaluated under reaction conditions by addressing the deactivation of the edges by hydrogen passivation or solid carbon formation. Although we found that the armchair edges were the most promising edge morphology for promoting the autocatalytic effect on CMD, due to the low free energy barrier needed for the desorption of H_2_, a high activation free energy is needed for the diffusion of H into the edges, being the rate determinant step for the dehydrogenation on the edges. The free energy of activation for hydrogen formation on the deposited carbon sites was higher in comparison with the reaction occurring on pristine armchair edges, showing that the H_2_ formation decreases during the CMD process, mainly due to the non-ordered growth of the armchair edges. With respect to the CMD steps on 12-ZGNR edges, we found an excellent performance for the methane decomposition, however, structural and electronic modification on the zigzag edges should be considered in order to increase the activity for H_2_ formation. We believe that the results presented in this work can provide fundamental insights for the design and synthesis of graphene-based catalysts for the activation and decomposition of methane and hydrogen production.

## Data Availability

Original datasets are available in a publicly accessible repository: The original contributions presented in the study are publicly available. This data can be found here: https://doi.org/10.15126/surreydata.900725.
