# Supplementary material for: Theoretical insights into the methane catalytic decomposition on graphene nanoribbons edges
Source: Front Chem. 2023 Jun 1;11:1172687. doi: 10.3389/fchem.2023.1172687 (PMC10267404; doi:10.3389/fchem.2023.1172687)
Supplement: Supplementary file 1 [file DataSheet1.pdf]

frontiers

Supplementary Material

Table S1. Adsorption energy for the d-CH_4, u-CH_4, t-CH_4 and u-CH_4-IP, obtained at the PBE-TS level (E$_{a}ds, TS$), PBE-MBD level (E$_{ads,MBD}$) and their energetic difference (ΔE_ads). Equilibrium distance between methane and the Z1 adsorption site, obtained at the PBE-TS level (D$_e, TS$), PBE-MBD level (D$_e, MBD$) and their difference (ΔD_e).

	E$_{ads,TS}$ (eV)	E$_{ads,MBD}$ (eV)	ΔE_{ads}	D$_{e,TS}$	D$_{e,MBD}$	ΔD_e
d-CH_4	-0.089	-0.070	0.019	2.750	2.960	0.210
u-CH_4	-0.120	-0.089	0.032	3.510	3.790	0.280
t-CH_4	-0.113	-0.072	0.041	3.610	3.950	0.340
u-CH_4-IP	-0.073	-0.070	0.003	2.810	2.800	-0.010

Table S2. Adsorption energies in eV for different orientations of CH_4 on Z1, Z2, Z3 and Z4 sites on the 12-ZGNR.

	Z1	Z2	Z3	Z4
u-CH_4	-0.120	-0.136	-0.180	-0.186
d-CH_4	-0.089	-0.115	-0.133	-0.136
t-CH_4	-0.113	-0.140	-0.177	-0.180

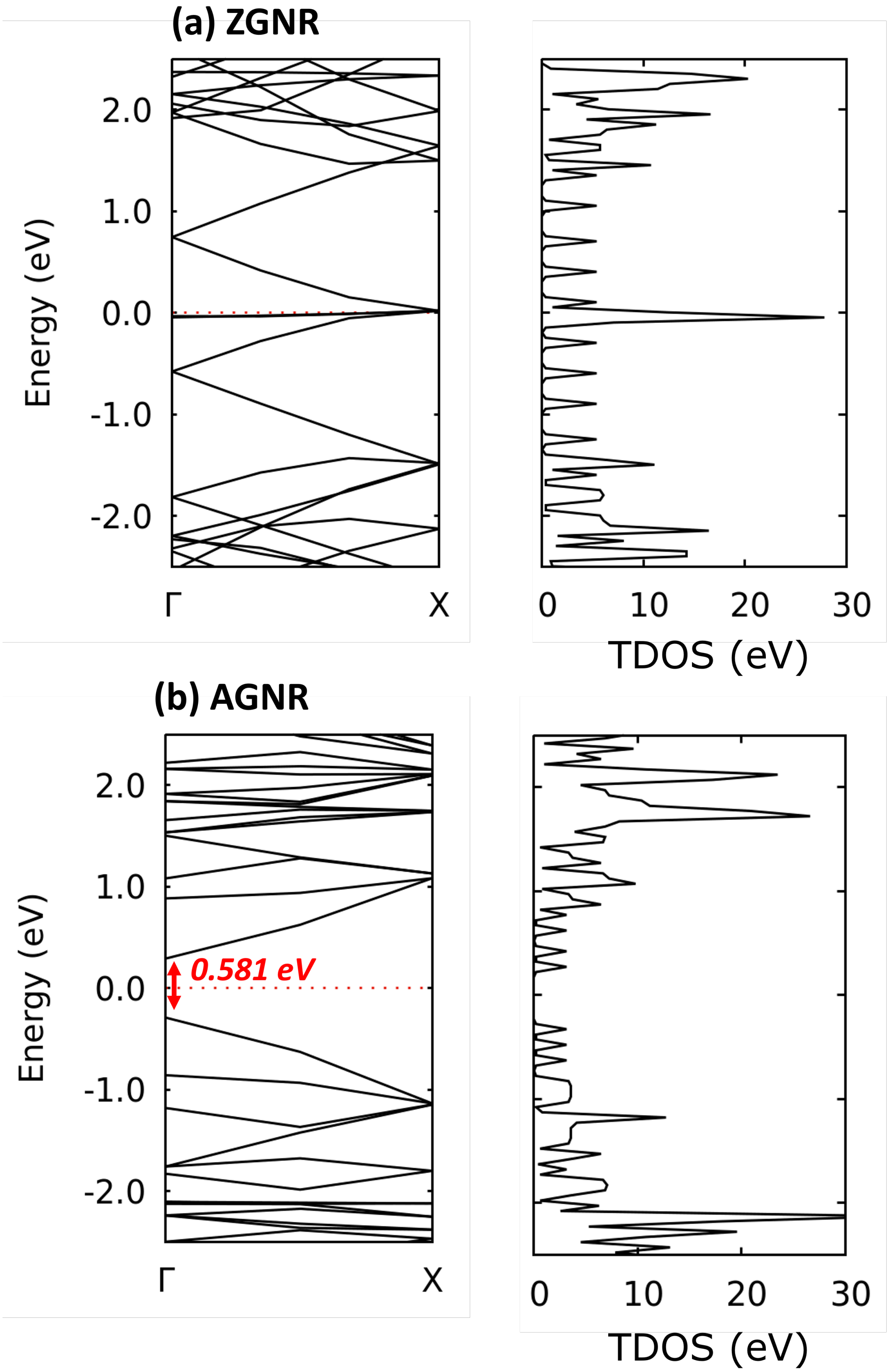

Figure S1. Band structure (left) and total density of states (right) of (a) pristine 12-ZGNR and (b) pristine 12-AGNR. A uniform (10 × 1 × 1) k-point sampling grid was adopted for the band structure calculations

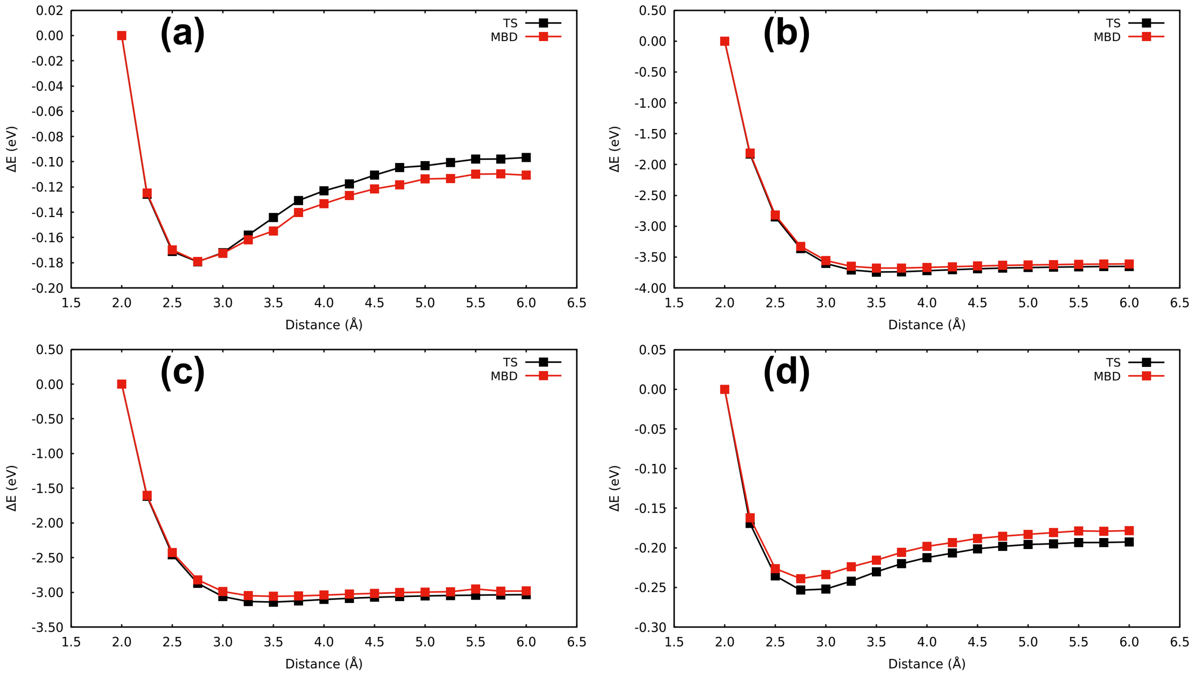

Figure S2. Potential energy surface as a function from the CH_4 distance from the Z1 adsorption site on 12-ZGNR, adopting the TS and MBD dispersion corrections. The PES were constructed in respect to the (a) d-CH_4 conformation; (b) t-CH_4 conformation; (c) u-CH_4 conformation physisorbed on Z1 site and (d) on the Z1-IP site.

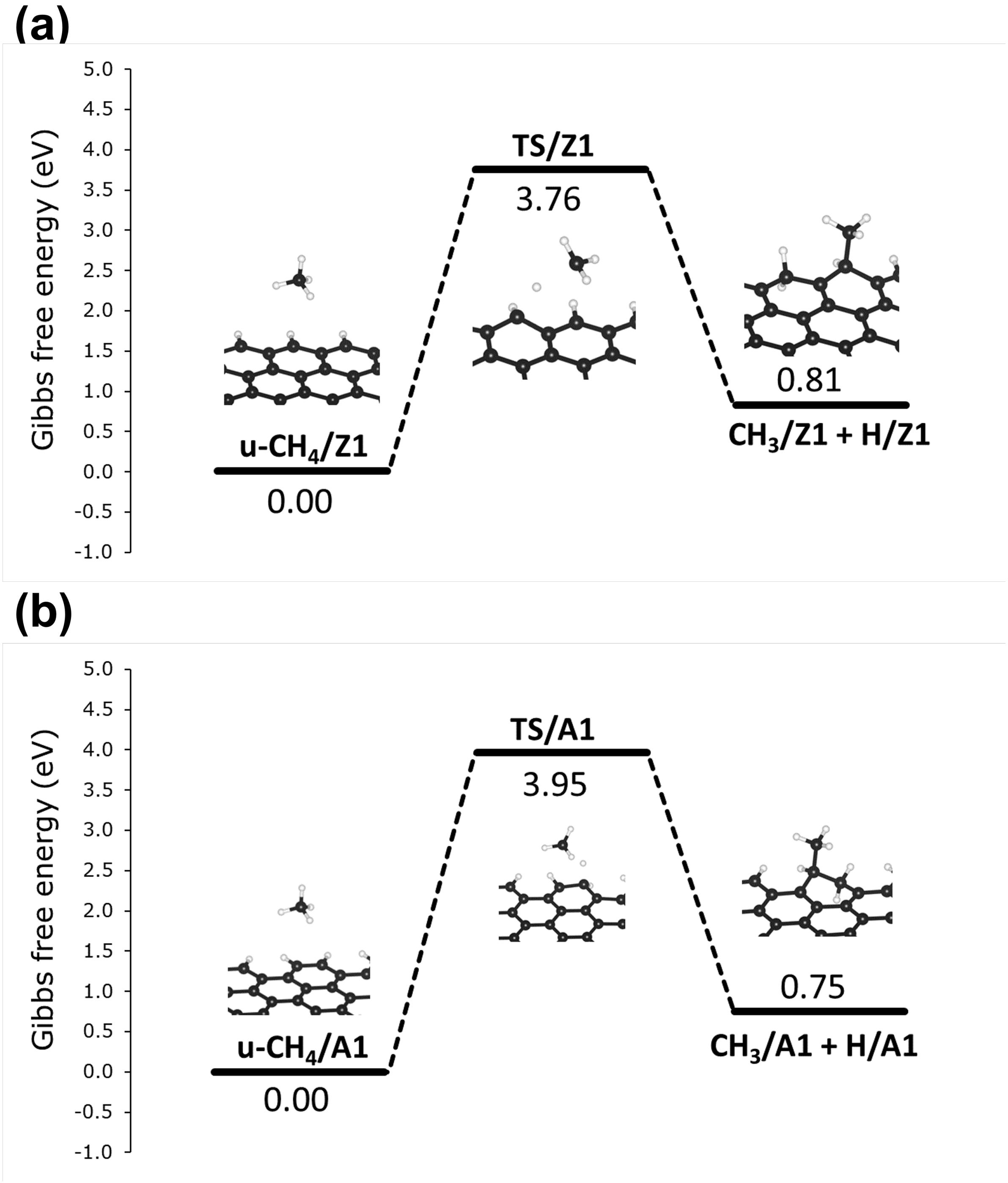

Figure S3. Gibbs free energy reaction profile of the u-CH_4 adsorption on the Z1 and A1 sites of the 12-ZGNR and 12-AGNR, respectively.

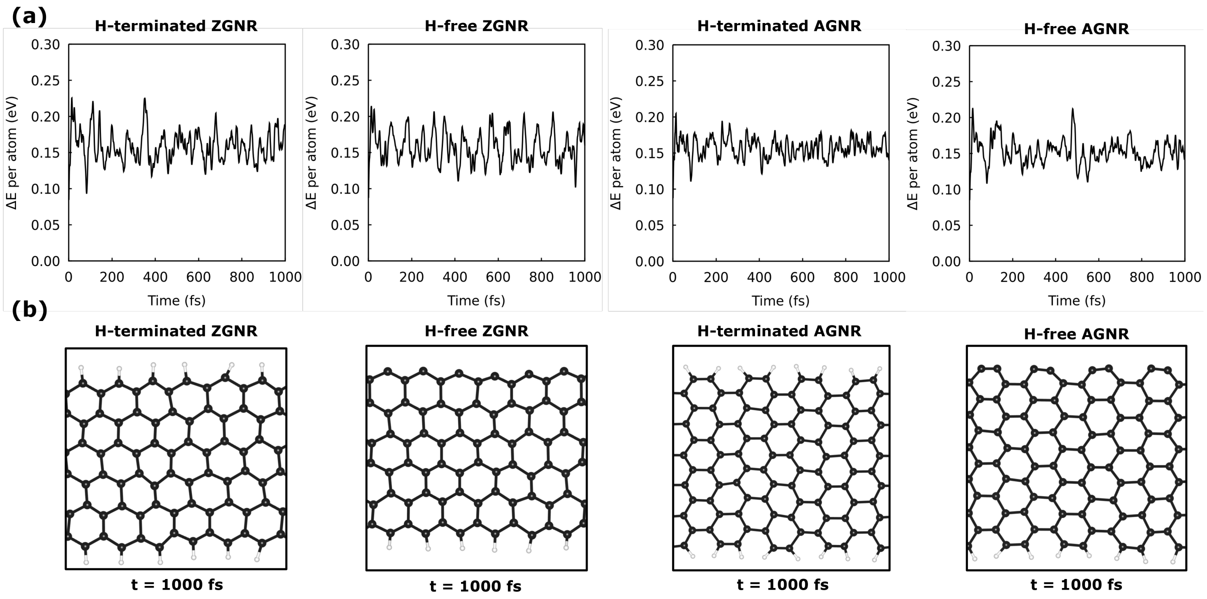

Figure S4. (a) Variation of electronic energy per atom, in respect to the minimum energy structure, against the time, upon AIMD simulations at 1200 K for the H-terminated and H-free ZGNR and AGNR (b) Snapshot of the each ZGNR and AGNR structures after 1000 fs simulation time.

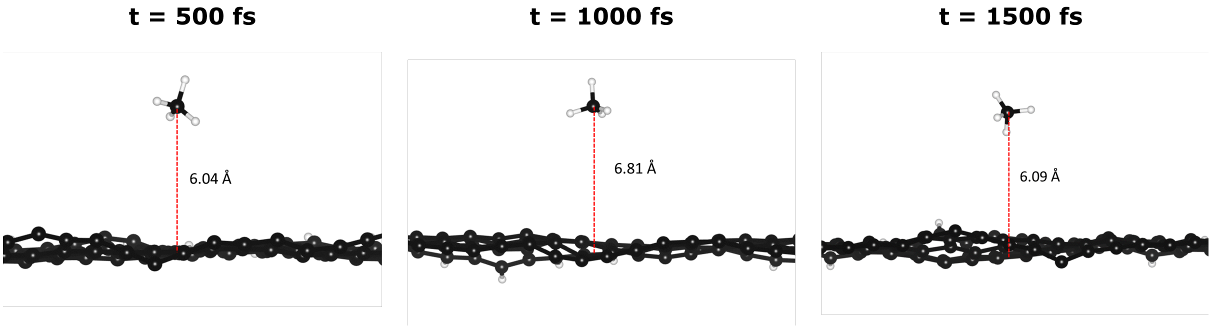

Figure S5. Snapshot of AIMD simulations of methane physisorbed on H-free ZGNR at 500 fs, 1000 fs and 1500 fs

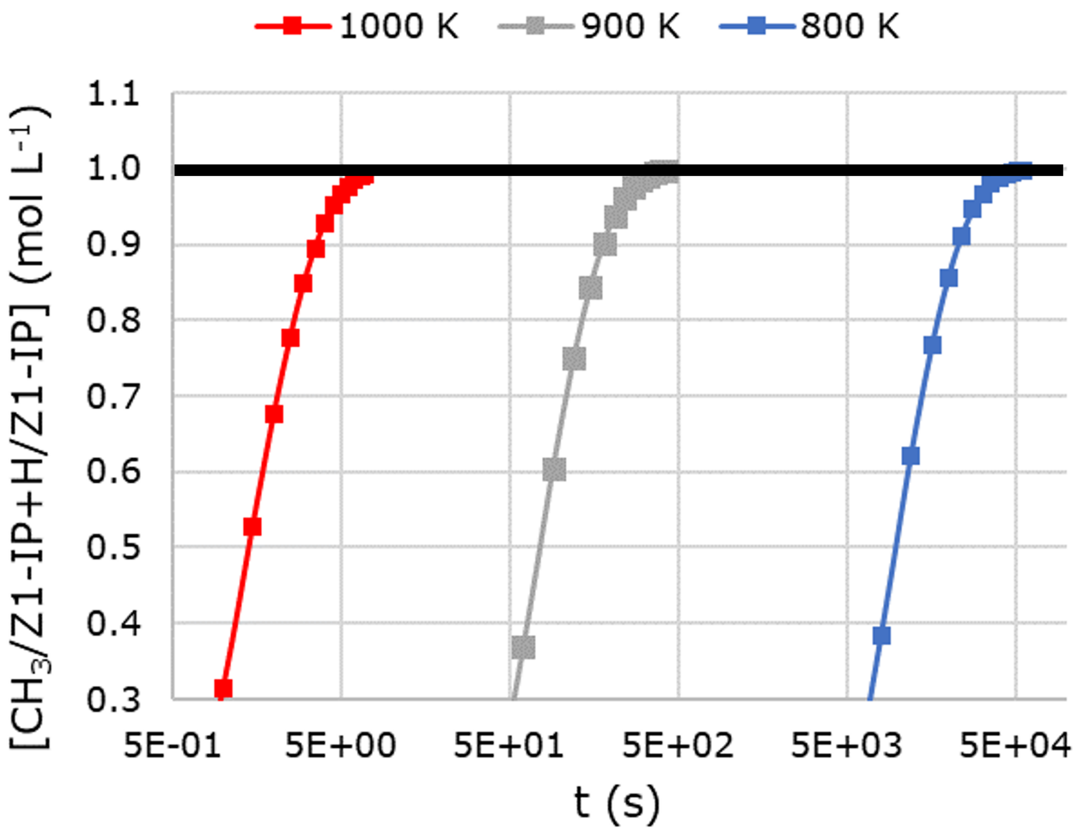

Figure S6. Concentration in mol L^{-1} as a function of time of the most thermodynamic favourable chemisorption product, CH_3/Z1 + H/Z1, from methane (u-CH_4/Z2). Rate coefficients were obtained by means of the Transition State Theory, at the temperatures of 800 K, 900 K and 1000 K. The lattice-gas thermodynamic model was adopted in all calculations. Simulations were made considering the reaction mechanisms (ii) and (iii) of the main manuscript, i.e., we considered u-CH_4/Z2 as the intermediate, and CH_3-H/Z_1 and CH_3/Z1 + H/Z1 as the initial and final states, respectively. Numerical simulations were done considering the steady-state approximation, in which d[u-CH_4/Z2]/dt $\approx$ 0.
